# Hypertransaminasemia Is a Marker of Severity in Children Hospitalized for Influenza

**DOI:** 10.1111/irv.70283

**Published:** 2026-06-24

**Authors:** Marco Poeta, Cristina Moracas, Elisabetta Venturini, Sofia Sgubbi, Maria Sole Valentino, Laura Folgori, Laura Petrarca, Claudio Cafagno, Marco Maglione, Anna Cascone, Claudia Colomba, Alice Romero, Fabio Midulla, Giuseppe Indolfi, Vania Giacomet, Amelia Licari, Luisa Galli, Alfredo Guarino

**Affiliations:** ^1^ Pediatric Infectious Disease Unit, Department of Maternal and Child Health University Hospital “Federico II” Naples Italy; ^2^ Department of Translational Medical Science University of Naples “Federico II” Naples Italy; ^3^ PhD National Programme in One Health Approaches to Infectious Diseases and Life Science Research, Department of Public Health, Experimental and Forensic Medicine University of Pavia Pavia Italy; ^4^ Infectious Diseases Unit, Meyer Children's Hospital Istituto di Ricovero e Cura a Carattere Scientifico (IRCCS) Florence Italy; ^5^ Department of Clinical, Surgical, Diagnostic and Pediatric Sciences Fondazione IRCCS Policlinico San Matteo Pavia Italy; ^6^ Pediatric Infectious Disease Unit, Luigi Sacco Hospital University of Milan Milan Italy; ^7^ Department of Paediatrics Vittore Buzzi Children's Hospital Milan Italy; ^8^ Department of Maternal, Infantile and Urological Sciences Sapienza University of Rome Rome Italy; ^9^ Infectious Diseases, Children's Hospital Giovanni XXIII Azienda Ospedaliero Universitaria Consorziale Policlinico di Bari Bari Italy; ^10^ Pediatric Emergency Department Santobono‐Pausilipon Children Hospital Naples Italy; ^11^ Residency School of Pediatrics, Department of Systems Medicine University of Rome “Tor Vergata” Rome Italy; ^12^ Infectious Disease Unit, Bambino Gesù Children's Hospital Istituto di Ricovero e Cura a Carattere Scientifico (IRCCS) Rome Italy; ^13^ Department of Health Promotion, Mother and Child Care, Internal Medicine and Medical Specialties University of Palermo Palermo Italy; ^14^ Department of Biomedical and Clinical Sciences University of Milan Milan Italy; ^15^ Meyer Children's Hospital Istituto di Ricovero e Cura a Carattere Scientifico (IRCCS) Florence Italy; ^16^ Department NEUROFARBA University of Florence Florence Italy; ^17^ Department of Health Sciences University of Florence Florence Italy

**Keywords:** aminotransferases, children, hypertransaminasemia, influenza, pediatric, prognostic value

## Abstract

**Purpose:**

Elevated transaminases have been associated with increased severity in adult influenza cases, but data in the pediatric population are limited. This study aims to evaluate the prevalence, clinical characteristics and prognostic value of elevated transaminases in children hospitalized for influenza.

**Methods:**

A multicentre retrospective cohort study was conducted on 543 children hospitalized for acute viral respiratory infections. Demographic, clinical, biochemical, radiological features, and outcome were collected and analyzed, comparing children with influenza to those with other respiratory viruses. The association between elevated transaminases and clinical severity, complications, and intensive care unit (ICU) admission was assessed.

**Results:**

Among 543 children, 127 (23.4%) had laboratory‐confirmed influenza, with 24.4% showing elevated transaminases versus 7.2% in noninfluenza infections (*p* < 0.001). Influenza B was associated with a higher prevalence of hypertransaminasemia (40%) than influenza A (20.6%, *p* = 0.042). Asthenia, myalgia, and hypovolemic shock were more common in the elevated transaminase group (*p* < 0.05). Biochemical markers indicated muscular rather than hepatic origin of hypertransaminasemia. Elevated transaminases correlated with length of hospital stay (10.2 vs. 5.8 days, *p* < 0.001), ICU admission (OR 4.4, *p* = 0.035), high‐flow oxygen need (OR 4.6, *p* = 0.005), and intravenous fluids (OR 6.2, *p* = 0.001). Two deaths occurred, both in the elevated transaminase group.

**Conclusion:**

Elevated transaminases occur in one fourth of children hospitalized for influenza and are associated with more severe disease, systemic complications, and worse outcomes. The findings suggest that transaminase elevation reflects systemic and muscular involvement rather than primary liver injury. Monitoring transaminases provides an early, easy marker to identify children at risk of severe influenza.

## 1 Introduction

Influenza infection affects people of all ages and is one of the leading causes of morbidity and mortality worldwide. Influenza viruses seasonally circulate and may cause a variety of diseases ranging from mild and self‐limiting upper respiratory symptoms to severe life‐threatening complications [1]. In Italy, 16 million cases of influenza‐like illness were reported during the 2024–2025 season. The season was characterized by the cocirculation of Influenza A and B viruses, with Type A (66.7%) predominating over Type B (33.3%) [2].

In childhood, influenza syndrome is one of the main causes of outpatient visits and hospital admissions and has a significant impact on healthcare systems [3]. Even though respiratory symptoms are the most frequent clinical feature of influenza, the disease can also extend beyond the respiratory tract, including the gastrointestinal system and causing symptoms such as diarrhea and vomiting. Extrapulmonary manifestations include life‐threatening complications such as encephalopathy, myositis, myocarditis, and acute renal failure [4].

Among the extrapulmonary manifestations, hypertransaminasemia has been increasingly reported. Despite a mild transient increase in aminotransferase levels being well documented in a number of viral infections, in influenza, this increase may be significant and associated with critical clinical outcomes [5].

In fact, hypertransaminasemia is part of the biochemical presentation of benign acute myositis in childhood (BACM) [6], but it is not limited to cases with clear myositis and may also interest patients without clinical or radiological signs of muscle involvement. In fact, observations in adults during the 2009 H1N1 pandemic showed an association between elevated AST and ALT levels and a higher risk of respiratory failure, intensive care admission, and mortality, even in previously healthy individuals [5].

However, the clinical significance of hypertransaminasemia is less clear in pediatric age [7]. Children often present with nonspecific or mild symptoms, so the identification of laboratory predictors of disease severity and progression is crucial to drive clinical decisions. In routine practice, AST and ALT elevations are frequently considered nonspecific or attributed to concomitant therapies, without a systematic evaluation of their prognostic value.

In this context, the aims of the present study were the definition of hypertransaminasemia prevalence in hospitalized influenza‐positive children and the identification of the role of aminotransferase increase as a potential biomarker of disease severity and progression in the pediatric population.

The study was initiated based on observations reported during meetings of the “Pediatric INF‐ACT Network,” an Italian collaborative network born to identify emerging infectious threats in the postpandemic era in childhood [8]. Severe cases of influenza associated with elevated transaminase levels raised attention during meetings, leading to the start of a multicenter analysis to characterize this unusual association.

## 2 Methods

### 2.1 Study Design and Patient Population

Pediatric patients (age 0–17 years) hospitalized with a flu‐like syndrome were enrolled in a multicenter retrospective study. Data were collected from January 2023 to May 2025 across 10 tertiary care centers of the “Pediatric INF‐ACT Network.” The network is part of the One Health Basic and Translational Actions Addressing Unmet Needs on Emerging Infectious Diseases (INF‐ACT) project, an EU‐funded National Recovery and Resilience Plan (NRRP) initiative with the aims of identifying emerging and re‐emerging infectious threats through monthly online meetings among clinicians from the 10 participating centers, which are representative of the Italian pediatric hospitalized population and geographically distributed from north to south of the country. The methodology has been used and described in previous studies published by the same network [8, 9, 10].

**FIGURE 1:**
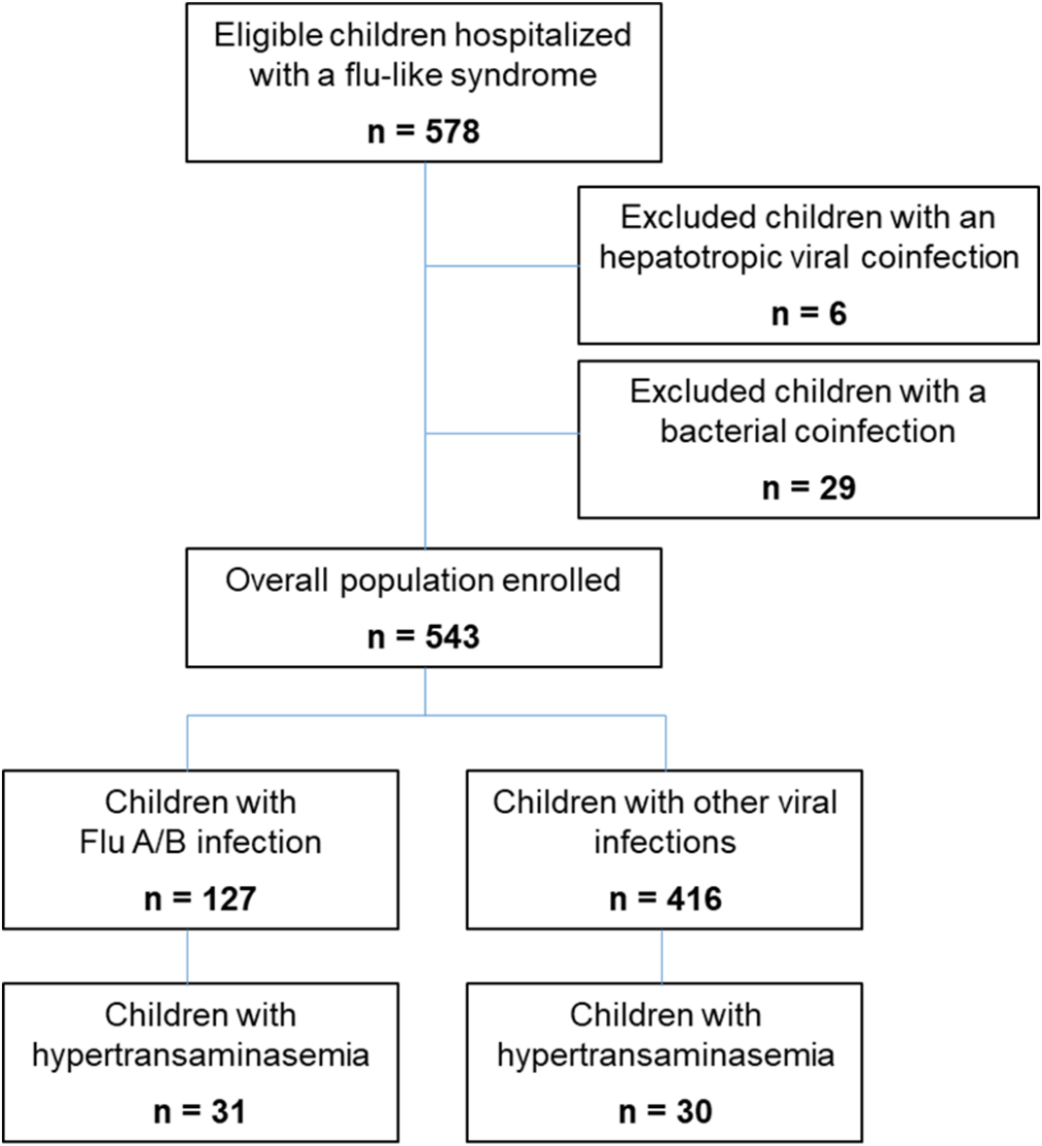
Study population.

The study design is illustrated in Figure 1. Hospitalized children were enrolled in presence of a flu‐like syndrome defined by fever associated with upper (e.g., rhinorrhea, cough, and sore throat) and/or lower respiratory tract manifestations (e.g., high respiratory rate, thorax retractions, nasal flaring, crackles or wheezing, and low oxygen saturation).

### 2.2 Group Stratification

Group stratification was based on nasopharyngeal swab detection of Influenza A or B viruses, assessed by Real Time‐Polymerase Chain Reaction (RT‐PCR), and the presence of hypertransaminasemia (i.e., AST and/or ALT levels greater or lower than twice the upper normal value [UNV] according to age).

The study group included children with at least one influenza‐positive sample. Influenza‐positive patients were further categorized into two groups based on their peak aminotransferase levels during hospitalization. Cases with a bacterial coinfection or a coinfection by hepatotropic viruses (e.g., Epstein–Barr virus and cytomegalovirus) were excluded due to the potential confounding effects on aminotransferase levels. Patients were not excluded based on underlying comorbidities, including immunocompromising conditions. Patients were not excluded based on underlying chronic comorbidities.

The frequency of hypertransaminasemia was additionally assessed in children with a flu‐like syndrome and a respiratory sample negative for influenza and positive for other respiratory viruses.

### 2.3 Data Collection

Data collection from electronic medical records included demographic characteristics (age, sex, ethnicity, presence of comorbidities, and influenza vaccination), clinical features (presenting symptoms, body temperature, and duration of fever), laboratory findings (complete blood count, inflammatory markers, and organ function biomarkers), radiological findings (presence of interstitial or lobar infiltrates and pleural effusion), and therapies (parenteral rehydration, antibiotics, antipyretic medications, oseltamivir, corticosteroids, bronchodilators, and oxygen need).

The comparative analysis between children with elevated aminotransferase levels and children with normal values was based on specific clinical and laboratory findings, including fever duration, inflammatory markers elevation, length of hospitalization, need for oxygen supplementation and corticosteroids therapy, admission to the intensive care unit (ICU), and in‐hospital mortality.

### 2.4 Statistical Analysis

All statistical analyses were performed using SPSS Statistics Version 29.0.1.0 (IBM Corp., Armonk, NY, United States). Descriptive statistics comprise counts + percentages (%) and mean ± standard deviation (SD) for categorical and continuous variables, respectively. Data were compared using the Chi‐square test, the Fisher's exact test, and the Student's *t* test, when appropriate. To investigate the factors associated with influenza severity, an univariable logistic regression was conducted with risk expressed as odds ratio (OR) and 95% confidence intervals (CIs). The level of significance was set at 0.05.

## 3 Results

### 3.1 Patient Characteristics and Prevalence of Hypertransaminasemia

A total of 578 children hospitalized with a flu‐like syndrome were initially enrolled in the study. Subsequently, 29 bacterial and six viral coinfections (one Epstein–Barr virus, one cytomegalovirus, two parvovirus B19, and two rotavirus) were excluded, given their potential direct role in aminotransferase levels increase. The remaining population of 543 children was further analyzed (313 males, 57.6%, mean age 43.0 ± 49.5 months).

**FIGURE 2:**
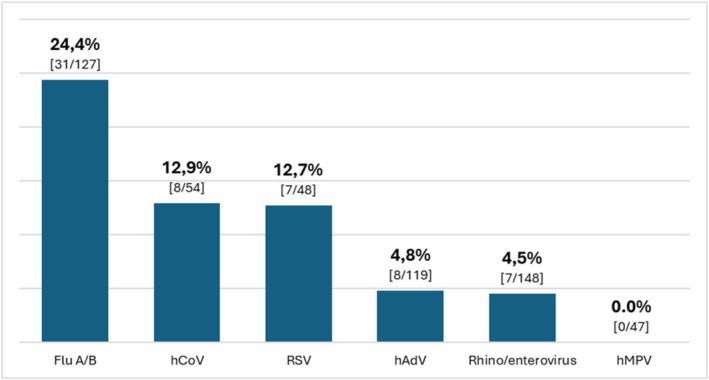
Frequency of hypertransaminasemia based on the viral pathogen identified by RT‐PCR from nasopharyngeal swab specimens. Abbreviations: Flu, influenza virus; hAdV, human adenovirus; hCoV, human coronavirus; hMPV, human metapneumovirus; RSV, respiratory syncytial virus. *Chi‐square test across different pathogens.

Among them, 127/543 children (23.4%) had a laboratory‐confirmed influenza infection (mean age 52.0 ± 52.9 months, 57.5% male), and 416/543 (76.6%) had a diagnosis of respiratory viral infection other than influenza (mean age 44.1 ± 54.3 months, 57.7% male). Comparing prevalence among the different viral etiologies, the frequency of hypertransaminasemia was significantly higher in influenza‐positive cases (24.4%, 31/127, vs. 7.2%, 30/416 *p* < 0.001) (Figure 2).

Influenza A and B were detected in 102/127 (80.3%) and 25/127 cases (19.7%), respectively. Hypertransaminasemia was more significantly reported in patients with Influenza B (40.0%, 10/25) than Influenza A infection (20.6%, 21/102) (*p* = 0.042). Fifty‐nine Influenza A cases had performed a subtype analysis showing: 53 H1N1 (89.8%), 4 H3N2 (6.8%), 1 H1N2 (1.7%), and 1 H1N3 (1.7%) cases. Hypertransaminasemia was reported in 13/53 H1N1 patients (24.5%) and in none of the other available subtypes.

Table 1 summarizes the demographic and clinical characteristics of the overall population. No significant differences were found in demographic features between the elevated and the normal aminotransferases groups. There were no significant differences in the prevalence of pre‐existing chronic comorbidities between the groups. No immunocompromised patients were identified in the overall population.

**TABLE 1:** Demographic and clinical features of overall population and children with Flu infection with or without aminotransferase increase.

	Overall population (*n* = 127)	AST or ALT > x 2 unv (*n* = 31)	AST or ALT < x 2 unv (*n* = 96)	*p*
Demographic features				
Male sex, *n* (%)	73 (57.5)	17 (54.8)	56 (58.3)	0.835
Non‐Caucasian, *n* (%)	24 (18.9)	6 (19.4)	18 (18.8)	0.473
Mean age, months (SD)	52.0 (52.9)	63.1 (51.7)	50.6 (55.6)	0.265
At least one comorbidity, *n* (%)	30 (23.8)	10 (32.3)	20 (21.1)	0.229
At least one coinfection, *n* (%)	38 (29.9)	3 (9.7)	35 (36.5)	0.006
Flu vaccination, *n* (%)	11 (8.6)	2 (6.5)	9 (9.4)	0.728
Flu A, *n* (%)	102 (80.3)	21 (67.7)	81 (84.4)	0.042
Flu B, *n* (%)	25 (19.7)	10 (32.3)	15 (15.6)	0.042
Clinical features				
Peak body temperature,°C mean (SD)	38.5 (3.4)	39.0 (0.8)	38.3 (4.2)	0.239
Fever duration, days (SD)	4.6 (3.8)	3.9 (2.0)	4.8 (4.4)	0.147
Cough, *n* (%)	77 (60.6)	16 (51.6)	61 (63.5)	0.292
Respiratory distress, *n* (%)	36 (28.3)	9 (29.0)	27 (28.1)	0.546
SpO2, % mean (SD)	95.9 (4.7)	95.7 (4.5)	95.9 (4.9)	0.857
Wheezing, *n* (%)	11 (8.7)	2 (6.5)	9 (9.5)	0.462
Poor feeding, *n* (%)	54 (42.5)	15 (48.4)	39 (40.6)	0.532
Sore throat, *n* (%)	27 (21.3)	5 (16.1)	22 (22.9)	0.614
Asthenia, *n* (%)	40 (31.5)	17 (54.8)	23 (24.0)	0.002
Myalgia, *n* (%)	9 (7.1)	5 (16.1)	4 (4.2)	0.039
Headache, *n* (%)	11 (8.7)	4 (12.9)	7 (7.3)	0.461
Conjunctivitis, *n* (%)	8 (6.3)	2 (6.5)	6 (6.3)	0.625
Vomiting, *n* (%)	19 (15.1)	7 (23.3)	12 (12.5)	0.155
Diarrhea, *n* (%)	17 (13.4)	9 (29.0)	8 (8.3)	0.008
Abdominal pain, *n* (%)	10 (7.9)	6 (19.4)	4 (4.2)	0.014
Rash, *n* (%)	7 (5.5)	2 (6.5)	5 (5.2)	0.678
Neurological symptoms, *n* (%)	31 (24.4)	6 (19.4)	25 (26.0)	0.310
Febrile seizures, *n* (%)	23 (18.1)	4 (12.9)	19 (19.8)	0.592
Acute encephalopathy, *n* (%)	6 (4.7)	3 (9.7)	3 (3.1)	0.283
Lymphadenopathy, *n* (%)	15 (11.6)	4 (12.9)	11 (11.5)	0.759
Moderate‐to‐severe dehydration, *n* (%)	13 (10.2)	6 (19.4)	7 (7.3)	0.077
Hypovolemic shock, *n* (%)	4 (3.1)	3 (9.7)	1 (1.0)	0.045

Abbreviations: flu, Influenza virus; *n*, number; SD, standard deviation.

### 3.2 Clinical Manifestations

Respiratory signs and symptoms (i.e., cough, respiratory distress, and SpO_2_ levels) were comparable between the two groups, whereas hypertransaminasemia groups showed a significantly higher incidence of asthenia (54.8% vs. 24.0%, *p* = 0.002), myalgia (16.1% vs. 4.2%, *p* = 0.039), and hypovolemic shock (9.7% vs. 1.0%, *p* = 0.045). Gastrointestinal symptoms were also more frequently reported in the elevated group, with higher frequencies of diarrhea (29.0% vs. 8.3%, *p* = 0.008) and abdominal pain (19.4% vs. 4.2%, *p* = 0.014).

### 3.3 Biochemical and Radiological Findings

Table 2 shows the biochemical and radiological findings of the overall population and the comparative analysis between the two groups of children.

**TABLE 2:** Biochemical and radiological findings of overall population and children with Flu infection with or without aminotransferases increase.

	Overall population (*n* = 127)	AST or ALT > x 2 unv (*n* = 31)	AST or ALT < x 2 unv (*n* = 96)	*p*
Biochemical parameters, means (SD)				
Hemoglobin, g/dL	11.9 (1.7)	11.8 (1.5)	11.9 (1.8)	0.868
WBC, cells/μL	9885 (5861)	8821 (6530)	10,277 (5622)	0.272
Neutrophils, cells/μL	6215 (5166)	5410 (4471)	6497 (5428)	0.272
Neutropenia, *n* (%)	8 (6.3)	5 (16.1)	3 (3.1)	0.021
Lymphocytes, cells/μL	2677 (2489)	2520 (2512)	2762 (2620)	0.648
Lymphopenia, *n* (%)	50 (39.4)	15 (48.4)	35 (36.5)	0.292
Monocytes, cells/μL	786 (550)	705 (752)	800 (463)	0.527
Platelet count, × 10^3^ cells/μL	304 (173)	249 (211)	328 (163)	0.032
Thrombocytopenia, *n* (%)	6 (4.7)	6 (19.4)	0 (0.0)	0.001
CRP, mg/L	26.4 (46.3)	35.5 (60.4)	25.6 (43.7)	0.412
PCT, ng/mL	6.9 (30.0)	10.5 (18.9)	6.1 (33.9)	0.517
Fibrinogen, mg/dL	337.8 (187.2)	255.3 (87.2)	412.3 (180.0)	0.003
D‐Dimer, ng/mL	2972 (5516)	5119 (8620)	1606 (1363)	0.325
AST, U/L	251 (1145)	772 (2059)	38 (13)	0.003
ALT, U/L	146 (516)	500 (939)	22 (13)	< 0.001
Bilirubin, U/L	0.6 (1.1)	0.5 (0.4)	0.5 (1.3)	0.881
GGT, U/L	28.5 (50.3)	53.5 (78.3)	21.2 (37.6)	0.057
Creatinine, mg/dL	0.4 (0.2)	0.41 (0.19)	0.36 (0.20)	0.247
CK, U/L	686 (1794)	1656 (2879)	239 (502)	< 0.001
LDH, U/L	543 (1082)	1087 (2032)	346 (89)	0.008
Ferritin, μg/L	3231 (9464)	6581 (3205)	253 (231)	0.050
Viral respiratory coinfection, *n* (%)	37 (29.1)	3 (6.5)	34 (35.4)	0.006
Chest X‐ray, *n* (%)				
Abnormal findings	46/67 (68.7)	9/14 (64.3)	37/53 (69.8)	0.751
Interstitial	17/67 (25.4)	4/14 (28.6)	13/53 (24.5)	0.740
Lobar	35/67 (52.2)	5/14 (35.7)	30/53 (56.6)	0.231
Pleural effusion	6/67 (9.0)	1/14 (7.1)	5/53 (9.4)	0.633

Abbreviations: ALT, alanine aminotransferase; AST, aspartate aminotransferase; CK, creatine kinase; CRP, C‐reactive protein; *n*, number; PCT, procalcitonin; SD, standard deviation; WBC, white blood cells.

The muscle injury biomarkers creatine kinase (CK) (mean 1656 U/L vs. 239 U/L, *p* < 0.001) and lactate dehydrogenase (LDH) levels (1087 U/L vs. 346 U/L, *p* = 0.008) were significantly higher in hypertransaminasemia group. Whereas AST and ALT mean values were, by definition, significantly higher in the elevated group, liver cholestatic biomarkers (i.e., bilirubin and GGT mean values) were comparable between the two groups of influenza‐positive children (*p* > 0.05).

In addition, the elevated transaminase group reported lower platelet counts (249 × 10^3^ cells/μL vs. 328 × 10^3^ cells/μL, *p* = 0.032) and fibrinogen levels (255 vs. 412 mg/dL, *p* = 0.003), and higher neutropenia incidence (*p* < 0.05) and ferritin levels (6581 vs. 253 μg/L, *p* = 0.050), suggesting systemic inflammatory involvement of influenza infection in the presence of hypertransaminasemia.

The overall rate of viral respiratory coinfections was significantly lower in the group with hypertransaminasemia compared to those with normal transaminase levels (3/31 6.5% vs. 34/96 35.4%, *p* = 0.006).

No significant differences were observed in abnormal radiological findings (i.e., interstitial, lobar pattern, and pleural effusion) between the two groups (*p* > 0.05). Abdominal ultrasonography, performed in 15 children, including eight from the hypertransaminasemia group, did not show evidence of liver damage.

### 3.4 Therapeutic Interventions and Outcomes

Patients with hypertransaminasemia required higher therapeutic supports (Table 3).

**TABLE 3:** Therapies and outcome of overall population and children with Flu infection with or without aminotransferases increase.

	Overall population (*n* = 127)	AST or ALT > x 2 unv (*n* = 31)	AST or ALT < x 2 unv (*n* = 96)	*p*
Therapies				
Parenteral rehydration, *n* (%)	77 (60.6)	27 (87.1)	50 (52.1)	< 0.001
Antibiotics, *n* (%)	64 (50.4)	18 (58.1)	46 (47.9)	0.410
Systemic corticosteroids, *n* (%)	32 (25.2)	9 (29.0)	23 (24.0)	0.636
Bronchodilators, *n* (%)	27 (21.3)	6 (19.4)	21 (21.9)	0.750
Paracetamol, *n* (%)	117 (92.1)	28 (90.3)	89 (92.7)	0.688
Inhaled steroids, *n* (%)	31 (24.4)	9 (29.0)	22 (22.9)	0.481
Oxygen supplementation, *n* (%)	39 (30.2)	14 (42.4)	25 (26.0)	0.045
HFNC oxygen, *n* (%)	26 (20.5)	13 (41.9)	13 (13.5)	0.001
Oseltamivir, *n* (%)	36 (28.3)	9 (29.0)	27 (28.1)	0.546
Outcome				
Death, *n* (%)	2 (1.6)	2 (6.5)	0 (0.0)	0.058
Intensive care, *n* (%)	9 (7.1)	5 (16.1)	4 (4.2)	0.024
Length of hospital stay, mean (SD)	6.9 (6.6)	10.2 (12.2)	5.8 (3.5)	0.002

Abbreviations: *n*, number; SD, standard deviation.

Rates of parenteral rehydration (87.1% vs. 52.1%, *p* < 0.001), oxygen supplementation (42.4% vs. 26.0%, *p* = 0.045), and high‐flow nasal cannula (HFNC) oxygen therapy (41.9% vs. 13.5%, *p* = 0.001) were significantly higher in presence of hypertransaminasemia. Paracetamol administration was comparable (90.3% vs. 92.7%, *p* > 0.05). Information regarding the administration of other antipyretic agents (i.e., NSAIDs) was not available.

Outcomes were considerably worse for patients with hypertransaminasemia in terms of intensive care admissions rate (16.1% vs. 4.2%, *p* = 0.024), length of hospital stay (mean 10.2 days vs. 5.8 days, *p* = 0.002), and mortality (two deaths occurred within the elevated transaminase group).

### 3.5 Comparative Analysis of Laboratory Parameters

**FIGURE 3:**
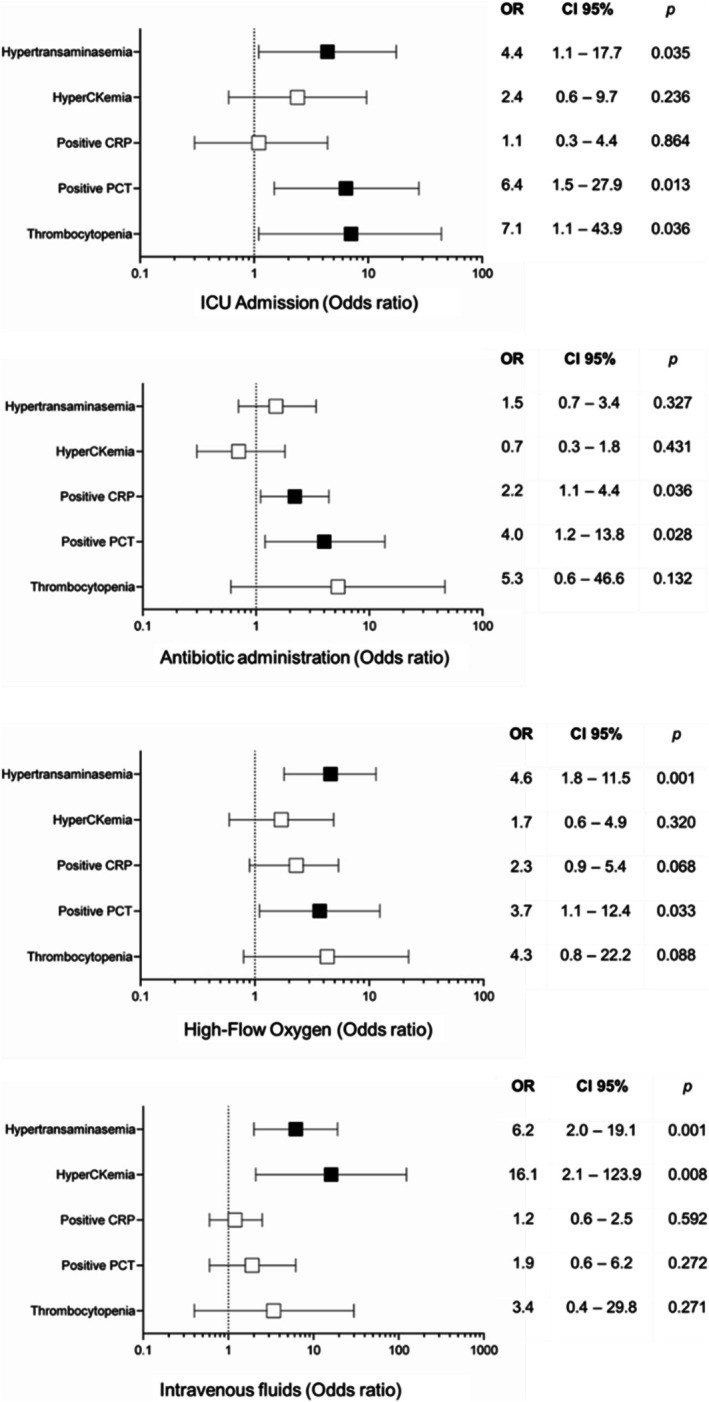
Therapeutic and severity outcomes based on laboratory marker alterations. Black squares indicate significant results; white squares indicate nonsignificant results. Abbreviations: CI, confidence interval; ICU, intensive care unit; OR, odds ratio.

Among laboratory abnormalities (Figure 3), the presence of hypertransaminasemia (twice the normal value for age) was a strong predictor of disease severity in terms of ICU admission (OR 4.4, *p* = 0.035), need for high‐flow oxygen (OR 4.6, *p* = 0.005), and parenteral rehydration (OR 6.2, *p* = 0.001). Among other laboratory markers, positive PCT was also correlated with ICU admission and the need for high‐flow oxygen, whereas both CRP and PCT were associated with antibiotic administration, unlike hypertransaminasemia.

## 4 Discussion

Elevated transaminase levels frequently occur during systemic infections, interesting approximately 10% of acute respiratory infections in children and adolescents [11]. In our cohort, a significantly higher percentage of influenza‐positive children had hypertransaminasemia compared to those infected with other respiratory viruses, suggesting a higher systemic involvement extending beyond the respiratory tract.

Hypertransaminasemia in influenza is often attributed to benign acute myositis in children (BACM) [6], but, in our cohort, we observed both in patients with muscle signs or symptoms (such as myalgia and increased CK) and in those without clear evidence of muscle involvement. Although the mechanisms of respiratory involvement are well defined, the pathogenesis of other organ damage is less clear [12]. In particular, transaminase elevation may result from both direct and indirect viral effects. A direct effect of influenza virus is well described; in fact, although the virus has never been isolated in muscle tissue, some viral antigens have been identified in muscle biopsies, suggesting a nonpermissive infection leading to fiber destruction and consequent release of muscle enzymes [13]. In addition, indirect effects due to systemic inflammation may contribute to muscle and liver injuries and include cytokine‐induced toxicity and tissue hypoperfusion [14].

In our cohort, muscle rather than liver involvement is the main factor contributing to hypertransaminasemia. Muscle enzyme levels (i.e., CK and LDH) were significantly higher in patients with elevated transaminases, whereas other liver markers (i.e., bilirubin and GGT) abdominal ultrasounds suggested no liver involvement. Nevertheless, the detailed pathogenesis of muscle damage in our population is unclear, as the relative contribution of direct viral and indirect immune‐mediated effects is unknown.

Furthermore, hypertransaminasemia was significantly more frequently observed in the group of children infected with Influenza B than in those infected with Subtype A, and within the latter, it was reported almost exclusively in cases of H1N1. This specific distribution of subtypes raises questions about the presence of different pathogenic mechanisms dependent on specific immune pathways and, consequently, different degrees of inflammation activated by specific influenza strains [15]. In fact, influenza H1N1 appears associated with high immune activation, which combined with hypoxia may lead to hepatocellular and muscular damages resulting in more severe clinical features and higher transaminase levels than other influenza subtypes [16].

Interestingly, whereas, in previous studies, lymphopenia had been related to disease severity [17], in our cohort, hypertransaminasemia was more frequently associated with neutropenia and thrombocytopenia, in line with recent findings of Lin et al. [18], who reported that children hospitalized with influenza had significantly lower white blood cell and platelet counts, along with elevated transaminase levels. The comparative analysis of laboratory parameters showed that elevated transaminases were most consistently associated with worse clinical outcomes, including longer hospital stays, greater need for supportive therapies (such as oxygen and fluid supplementation), ICU admissions, and mortality than other biomarkers. Notably, ICU admission was not associated with elevated CRP, unlike in children hospitalized with COVID‐19, where higher levels have been consistently associated with increased disease severity [19]. In contrast, despite the association with overall systemic severity and, in particular, with higher oxygen requirements in the elevated transaminase group, pulmonary alterations (i.e., consolidations, interstitial patterns, and pleural effusion) were similarly reported in both groups without statistical significance differences. Instead, children with hypertransaminasemia more frequently exhibited diarrhea and vomiting, resembling the clinical picture of Multisystem Inflammatory Syndrome of Children (MIS‐C), a COVID‐related complication characterized by systemic inflammation and gastrointestinal involvement [20]. In addition, four patients in the hypertransaminasemia group presented with GI symptoms accompanied by multiorgan involvement, characterized by hematological abnormalities in all cases and cardiac involvement, including elevated cardiac biomarkers and transient left ventricular dysfunction on echocardiography in two cases. These patients exhibited a hyperinflammatory phenotype with multisystem involvement that partially overlapped with the clinical features of MIS‐C, suggesting a possible common mechanism driven by systemic inflammation, rather than direct viral effects, capable in both cases of inducing immune‐mediated injury beyond the respiratory tract.

Respiratory viruses other than influenza virus, such as adenovirus and respiratory syncytial virus, may be associated with mild and transient increases in transaminases, particularly in younger or immunocompromised children [9, 16]. In our study, these viruses were more commonly detected in children without hypertransaminasemia, supporting the limited contribution of viral coinfections to transaminase elevation and the predominant role of influenza. In fact, influenza appears to be a single specific pathogen capable of inducing cellular damage, similar to what has been observed in adult studies, in which the increases in AST, ALT, and creatine kinase have been proposed as a laboratory biomarker distinctive of influenza etiology than other influenza‐like illnesses [5].

In the post‐COVID era, influenza infections are associated with greater clinical severity, prolonged hospitalizations, and a higher rate of complications in children [17]. This effect appears a direct consequence of the so‐called “immune debt,” which follows pandemic‐related restrictions resulting in a low circulation and, consequently, reduced exposure to common pathogens, including influenza [21]. In this context, the emergence of severe influenza cases associated with hypertransaminasemia has been reported as a potential infectious threat during meetings of the INF‐ACT pediatric network [8], highlighting the complementary role of clinical networks alongside virological and epidemiological surveillance systems in defining the real‐world impact of circulating pathogens.

Although antiviral therapy is recommended for all hospitalized patients with suspected or confirmed influenza [1], only 29% of our cohort received oseltamivir, with no difference between patients with or without high transaminases. We did not specifically study whether such treatment was effective in decreasing transaminases. However, considering the association between elevated transaminases and more severe clinical outcomes, this subgroup may particularly benefit from timely antiviral treatment, and our results highlight a potential adherence gap to the recommended treatments. In addition, the vaccination rate was also remarkably low, in line with national coverage for the pediatric population [22], and the vaccination status did not differ between the two groups. Considering the possibility of severe manifestations of the infection in children, our data underscore the urgent need to strengthen influenza vaccination campaigns [23].

Study limitations include the retrospective design and the absence of a definitive diagnosis of myositis, as muscle biopsies and advanced imaging techniques were not performed. Furthermore, although no differences in paracetamol use were observed between groups, details on the number of administrations and drug doses were not available, and therefore, a potential role of overdose cannot be excluded. However, given the similar percentages reported in the two groups of children and the muscular origin of transaminases increase, paracetamol seems to play a negligible role.

Future prospective studies involving larger cohorts are needed to validate these findings and longitudinal follow‐up of children with hypertransaminasemia may clarify the potential risk of long‐term sequelae. Furthermore, although the elevation of transaminases appears to be directly associated with systemic inflammation induced by influenza infection [12], environmental factors (i.e., diet, lifestyle, and microbiota composition) should also be considered within a One Health perspective and multinational studies, including non‐Western settings, to define the global relevance of our findings.

In conclusion, hypertransaminasemia is a frequent feature of pediatric influenza infection and may serve as an early warning sign (“red flag”) to identify patients at increased risk for severe course and complications. Their integration into routine clinical assessment could support earlier and more tailored management decisions, including prolonged observation, timely start of antiviral therapies, or referral to higher level care facilities. For these reasons, the inclusion of AST and ALT values into risk stratification tools or clinical severity scores may facilitate decision‐making processes, particularly during seasonal influenza outbreaks.

## Author Contributions

**Marco Poeta:** conceptualization, methodology, data curation, investigation, validation, formal analysis, supervision, visualization, project administration, writing – original draft, writing – review and editing. **Cristina Moracas:** conceptualization, methodology, data curation, investigation, validation, formal analysis, project administration, writing – original draft, writing – review and editing, software. **Elisabetta Venturini:** data curation, investigation, writing – review and editing, validation. **Sofia Sgubbi:** data curation, investigation, validation, writing – review and editing. **Maria Sole Valentino:** investigation, validation, data curation, writing – review and editing. **Laura Folgori:** investigation, validation, data curation, writing – review and editing. **Laura Petrarca:** data curation, validation, investigation, writing – review and editing. **Claudio Cafagno:** validation, investigation, data curation, writing – review and editing. **Marco Maglione:** investigation, validation, data curation, writing – review and editing. **Anna Cascone:** investigation, validation, data curation, writing – review and editing. **Claudia Colomba:** writing – review and editing, supervision. **Alice Romero:** investigation, validation, data curation, writing – review and editing. **Fabio Midulla:** writing – review and editing, supervision, validation. **Giuseppe Indolfi:** writing – review and editing, supervision, validation. **Vania Giacomet:** writing – review and editing, supervision, validation. **Amelia Licari:** writing – review and editing, supervision. **Luisa Galli:** writing – review and editing, supervision, validation. **Alfredo Guarino:** writing – review and editing, resources, project administration, supervision, conceptualization, methodology, validation, funding acquisition.

## Funding

This research was supported by the EU funding within the Next Generation EU‐MUR PNRR Extended Partnership Initiative on Emerging Infectious Diseases (Project No. PE00000007, INF‐ACT).

## Ethics Statement

The study protocol was approved by the Ethics Committee for Biomedical Activities, University of Naples Federico II, Naples, Italy (Protocol Number 76/2023), and conducted in accordance with the Declaration of Helsinki. Written informed consent was obtained from the parents of the children involved in the study.

## Conflicts of Interest

The authors declare no conflicts of interest.

## Data Availability

The data that support the findings of this study are not openly available due to reasons of sensitivity and are available from the corresponding author upon reasonable request. Data are located in controlled access data storage at University Federico II of Naples.
